# Tobacco smoking and its impact on pain intensity of temporomandibular disorders: A systematic review and metanalysis

**DOI:** 10.1111/joor.13845

**Published:** 2024-09-09

**Authors:** Amarshree A. Shetty, Sultan Abdulrahman Almalki, AlBandary Hassan Al Jameel, Inderjit Murugendrappa Gowdar, Vincenzo Ronsivalle, Marco Cicciù, Giuseppe Minervini

**Affiliations:** ^1^ Department of Paediatric and Preventive Dentistry A.B Shetty Memorial Institute of Dental Sciences, NITTE (Deemed to Be University) Mangalore Karnataka India; ^2^ Department of Preventive Dental Sciences, College of Dentistry Prince Sattam Bin AbdulAziz University Al‐kharj Saudi Arabia; ^3^ Department of Periodontics and Community Dentistry, College of Dentistry King Saud University Riyadh Saudi Arabia; ^4^ Department of Biomedical and Surgical and Biomedical Sciences Catania University Catania Italy; ^5^ Saveetha Dental College and Hospitals, Saveetha Institute of Medical and Technical Sciences (SIMATS) Saveetha University Chennai Tamil Nadu India; ^6^ Multidisciplinary Department of Medical‐Surgical and Dental Specialties University of Campania Luigi Vanvitelli Naples Italy

**Keywords:** orofacial pain, pain intensity, temporomandibular disorders, tobacco smoking

## Abstract

**Background:**

Temporomandibular disorders (TMDs) encompass a spectrum of orofacial conditions characterised by pain and dysfunction in the temporomandibular joint and surrounding structures. Tobacco smoking has been posited as a potential factor influencing the prevalence and intensity of TMD. However, the nature and extent of this relationship remain unclear due to variations in study outcomes. This systematic review aimed to consolidate existing research findings to elucidate the association between tobacco smoking and TMD pain intensity.

**Methods:**

A comprehensive search of electronic databases was conducted to identify relevant studies published up to June 2023. Studies investigating the relationship between tobacco smoking and TMD pain were included. Data extraction was conducted by two reviewers. Quality assessment was performed using the New Castle‐Ottawa scale. Review Manager 5.4 was used to quantitatively analyse the results.

**Results:**

The review included four studies employing similar TMD assessment techniques. All studies reported elevated TMD pain intensity among tobacco users, with non‐smokers exhibiting lower pain intensity. The quality of the included studies was good. Meta‐analytic results showed that TMD pain intensity was higher in the smokers group compared to the non‐smokers group, with a weighted mean difference (WMD) of 0.65 (BPM) (95% CI: [0.10, 1.19], *p* = .02).

**Conclusion:**

This systematic review provides a comprehensive synthesis of the existing literature on tobacco smoking and TMD symptoms. The findings underscore the multifaceted nature of the relationship between smoking and TMD pain, highlighting its clinical relevance and the need for tailored interventions. Further research is warranted to elucidate underlying mechanisms and potential moderating factors, contributing to a more nuanced understanding of this complex association.

## Graphical abstract

Tobacco smoking significantly increases the pain intensity in patients with temporomandibular disorders (TMD): A systematic review and meta‐analysis reveals the detrimental effects of smoking on TMD pain, highlighting the need for targeted interventions in smoking cessation and TMD management.

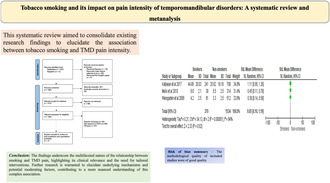

## 1 INTRODUCTION

Temporomandibular disorders (TMDs) constitute a complex and multifactorial group of orofacial conditions characterised by pain and dysfunction in the temporomandibular joint (TMJ) and surrounding structures.^1^ These conditions encompass a range of signs and symptoms, including orofacial pain, joint sounds, limited jaw mobility and associated musculoskeletal discomfort. TMD represents a significant public health concern due to its prevalence and the substantial impact it has on individuals' quality of life, encompassing aspects such as eating, speaking, and overall well‐being (Table 1). Smoking is also one of the causes of prosthesis failure.^1^, ^2^, ^3^, ^4^, ^5^, ^6^, ^7^, ^8^

**TABLE 1:** Abbreviations used in this review.

Term	Abbreviation used
Temporomandibular disorders	TMDs
Temporomandibular joint	TMJ
Current smokers	CS
Former smokers	FS
Non‐smokers	NS
Chronic Pain Grading Scale	CPGS
Odds ratio	OR

The use of tobacco products has long been acknowledged as a major risk factor for a number of chronic illnesses, such as cardiovascular disorders, chronic obstructive pulmonary disease and many types of cancer.^9^ Recently, its connections to osteoporosis and periodontal disease have also come under the spotlight. Notably, research has looked at the connection between smoking tobacco and chronic pain, with an emphasis on musculoskeletal sites, particularly the neck and lower back areas.^9^, ^10^, ^11^, ^12^ Surprisingly, little effort has been put into determining how smoking affects conditions other than the lower back.

Smoking tobacco is a widely acknowledged health risk factor that is mostly linked to respiratory and cardiovascular conditions, different malignancies and problems with dental health.^12^ However, the connection between tobacco usage and TMD continues to be a topic of intense interest and research in the fields of dentistry and orofacial pain.^12^, ^13^, ^14^, ^15^, ^16^, ^17^, ^18^, ^19^ Although this link has been the subject of numerous studies, the results have been inconsistent, leading to no firm conclusions. It is important for both clinical and public health reasons to comprehend how tobacco use may affect the frequency and severity of TMD. Understanding this connection can help preventive measures, improve patient treatment, and direct efforts to lessen the burden of TMD in tobacco‐exposed populations. The purpose of this systematic review was to analyse and synthesise all available data on tobacco use's potential impact on the frequency and severity of pain due to TMDs.

## 2 MATERIALS AND METHODS

### 2.1 Eligibility criteria

The review adhered to the PRISMA protocol^20^ so as to assure transparency and rigour in the review process by providing a strict, standardised structure.

The PECO (population, exposure, comparison and outcome) protocol for this investigation was defined to guide the selection process of articles.

Population (P): The target population consisted of human individuals.

Exposure (E): The exposure of interest in this review was the use of smoking tobacco. Only current smokers were considered. The exposure group consisted of individuals who were current smokers, encompassing a range of smoking habits and durations.

Comparison (C): The comparison group comprised individuals who were non‐smokers. This included those who had never smoked. The comparison group served as a reference to assess the differences in the prevalence and intensity of TMDs between smokers and non‐smokers.

Outcome (O): The primary outcome of interest in this review was the prevalence and intensity of TMD pain among individuals who smoke (current smokers) compared to those who do not smoke (non‐smokers). Prevalence was defined as the proportion of TMD cases within the population of smokers, while intensity referred to the severity and impact of TMD pain symptoms experienced by smokers compared to non‐smokers.

Clinical studies that explored the association between tobacco smoking and TMDs were included. Participants of all ages and genders diagnosed with TMD were considered eligible. There were no restrictions placed on the publication timeline or demographic characteristics. Peer‐reviewed articles published only in English were included. Animal studies, case studies and editorials were excluded. Non‐peer‐reviewed sources such as conference abstracts, dissertations and grey literature were also not included.

### 2.2 Search strategy

The database search protocol was meticulously designed to ensure a comprehensive and systematic retrieval of relevant studies, as seen in Table 2. Eight different electronic databases were searched using MeSH (Medical Subject Headings) keywords incorporated with Boolean operators (AND, OR). For each database, a customised search string was created to accommodate the specific indexing and controlled vocabulary of that database.

**TABLE 2:** Search strings across the utilised databases.

Database	Search String
PubMed	(“Temporomandibular Joint Disorders”[Mesh] OR “Temporomandibular Joint Dysfunction Syndrome”[Mesh] OR “TMD” OR “Temporomandibular Disorder*” OR “TMJ Disorder*”) AND (“Smoking”[Mesh] OR “Tobacco Smoking”[Mesh] OR “Cigarette Smoking”[Mesh] OR “Tobacco Use”[Mesh] OR “Tobacco”[Mesh] OR “Smoker*”) AND (“Prevalence”[Mesh] OR “Incidence”[Mesh] OR “Epidemiology”[Mesh])
Embase	(‘Temporomandibular Joint Disorders’/exp OR ‘Tobacco Smoking’/exp) AND (‘Prevalence’/exp OR ‘Incidence’/exp)
CINAHL	(“Temporomandibular Joint Disorders”[Mesh] OR “Temporomandibular Joint Dysfunction Syndrome”[Mesh] OR “TMD” OR “Temporomandibular Disorder*” OR “TMJ Disorder*”) AND (“Tobacco Use Cessation”[Mesh] OR “Smoking Cessation”[Mesh] OR “Smoking”[Mesh] OR “Tobacco Smoking”[Mesh] OR “Tobacco Use”[Mesh]) AND (“Incidence”[Mesh] OR “Prevalence”[Mesh] OR “Epidemiology”[Mesh])
Web of Science	(TS = (“Temporomandibular Disorders” OR “Temporomandibular Joint Dysfunction” OR “TMD”) AND TS = (“Smoking” OR “Tobacco Smoking” OR “Cigarette Smoking”)) OR (TS = (“Temporomandibular Disorders” OR “Temporomandibular Joint Dysfunction” OR “TMD”) AND TS = (“Prevalence” OR “Incidence” OR “Epidemiology”))
Scopus	(TITLE‐ABS‐KEY (“Temporomandibular Disorders” OR “Temporomandibular Joint Dysfunction” OR “TMD”) AND TITLE‐ABS‐KEY (“Smoking” OR “Tobacco Smoking” OR “Cigarette Smoking”)) OR (TITLE‐ABS‐KEY (“Temporomandibular Disorders” OR “Temporomandibular Joint Dysfunction” OR “TMD”) AND TITLE‐ABS‐KEY (“Prevalence” OR “Incidence” OR “Epidemiology”))
PsycINFO	(TI = (“Temporomandibular Disorders” OR “Temporomandibular Joint Dysfunction” OR “TMD”) AND TI = (“Smoking Behaviour” OR “Tobacco Dependence” OR “Oral Health”)) OR (TI = (“Temporomandibular Disorders” OR “Temporomandibular Joint Dysfunction” OR “TMD”) AND TI = (“Prevalence” OR “Incidence” OR “Epidemiology”))
AMED (Allied and Complementary Medicine Database)	(“Temporomandibular Joint Disorders”[Mesh] OR “Temporomandibular Joint Dysfunction Syndrome”[Mesh] OR “TMD” OR “Temporomandibular Disorder*” OR “TMJ Disorder*”) AND (“Smoking”[Mesh] OR “Tobacco”[Mesh] OR “Tobacco Smoking”[Mesh] OR “Tobacco Use”[Mesh]) AND (“Prevalence”[Mesh] OR “Incidence”[Mesh] OR “Epidemiology”[Mesh])
ERIC (Education Resources Information Center)	(“Temporomandibular Joint Disorders” OR “Temporomandibular Joint Dysfunction” OR “TMD”) AND (“Smoking Behaviour” OR “Tobacco Dependence” OR “Health Surveys”)

### 2.3 Data extraction

The data extraction included the assessment of variables such as study ID, publication year, study design, geographic location, sample size, age of participants and gender distribution. Technical data elicited details of the TMD evaluation technique, pain scale assessment, inferences observed and follow‐up period. Two independent reviewers extracted data from the included studies. To assess the reliability of data extraction, an interrater reliability test was conducted using Cohen's Kappa statistic. Any discrepancies in data extraction were addressed through discussion and consensus.

### 2.4 Risk of bias in evaluation

For assessing the bias in the selected papers for this review, the Newcastle‐Ottawa Scale (NOS) was employed.^21^ This tool assesses the quality of non‐randomised studies and assigns a score based on three domains. The NOS assigns a maximum of 9 stars to each study: 4 stars for the selection domain, 2 stars for the comparability domain and 3 stars for the exposure or outcome domain. A higher star rating indicates better methodological quality.

### 2.5 Statistical analysis

Review Manager 5.4^22^ was used for synthesising quantitative analysis. A random effect model was applied, considering the variability of the studies. The odds ratio at a 95% confidence interval was calculated. Forest plots were drawn to visualise the difference between the smokers group and the non‐smokers group for TMD pain intensity.

## 3 RESULTS

### 3.1 Study selection

**FIGURE 1:**
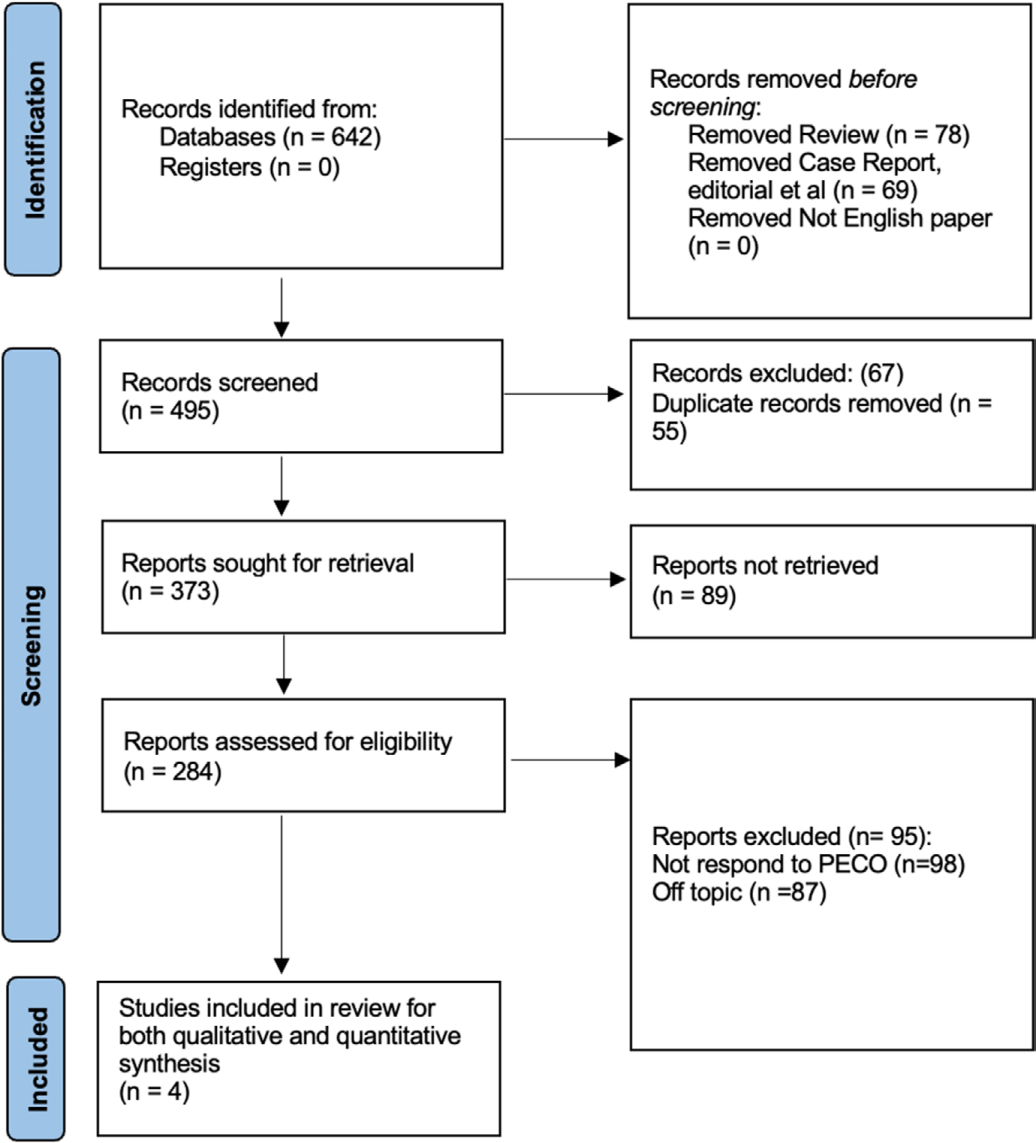
Study selection process for this review.

A comprehensive search of the literature yielded 642 records. Out of which, 55 duplicates were removed, leaving 587 papers to be screened. Of the total 137 reports in the final assessment, after excluding ineligible articles, only four studies^23^, ^24^, ^25^, ^26^ were included in the final qualitative review, as seen in Figure 1.

### 3.2 Study characteristics

The four studies included in this synthesised review were published between 2009 and 2017. Three studies were from the USA,^24^, ^25^, ^26^ while one was from India.^23^ The sample size ranged from 352 to 962. There were no age restrictions in the population studied, as tobacco use is prevalent across all age groups. Three studies employed a prospective study design,^23^, ^24^, ^25^ while one followed a retrospective study design.^26^ In all studies, the proportion of females significantly outweighed males. This gender skew may have relevance, as TMD is known to affect females more frequently than males, and gender can potentially interact with the impact of smoking on TMD symptoms. (Table 3).

**TABLE 3:** Demographic characteristics pertaining to the included papers.

Author	Year	Region assessed	Sample size (*n*)	Age (in years)	Gender ratio	Protocol
Katyayan et al.^23^	2017	India	962	31–50	599 females	Prospective
Melis et al.^24^	2010	USA	352	All ages	264 females	Prospective
Sanders et al.^25^	2012	USA	299	18–60	All females	Prospective
Weingarten et al.^26^	2009	USA	606	42.5 ± 15	497 females	Retrospective

### 3.3 Main findings

Table 4 represents a comprehensive overview of findings from the included studies^23^, ^24^, ^25^, ^26^ that collectively investigate the relationship between tobacco smoking and TMDs. All studies evaluated TMD symptoms using Research Diagnostic Criteria for Temporomandibular Disorders (RDC/TMD). Pain was assessed using Visual Analogue Scale (VAS),^23^ Numeric Rating Scale (NRS),^24^ and Clinical Pain Grade Scale (CPGS).^26^ The follow‐up period was for 6 months, as backed up by the International Association for the Study of Pain (IASP) Task Force on Taxonomy for Pain. Pain intensity was severe in all the studies in the ‘current smokers’ group as compared to ‘no smokers’.

**TABLE 4:** Characteristics pertaining to TMD pain intensity and tobacco smoking as observed in the selected papers.

Study	Assessment technique	Pain asssessment technique	Inferences	Follow up period
Katyayan et al.^23^	RDC/TMD	VAS (100 gauge)	VAS score in YS was 44.89 + 20.03 as compared to 25.82 + 16.16	6 months
Melis et al.^24^	RDC/TMD	Numeric Rating scale (0–10 gauge)	Pain scores in YS was 6.6 + 2.1 as compared to 5.5 ± 2.5 in NS. Greater pain intensity was noted in subjects who smoked larger numbers. A significant positive correlation was also noted among females (*p* = .001)	6 months
Sanders et al.^25^	RDC/TMD	Not specified	YS had 3.3 OR of TMD risk as compared to NS Older age had greater odds of TMD Other significant risk factors were allergies, IL—1 antagonist and anxiety score	6 months
Weingarten et al.^26^	RDC/TMD	Chronic Pain Grade Scale	Pain in YS population was 4.2 ± 2.5 as compared to 3.3 ± 2.5, significant at *p* = .01 in the adjusted model	Not applicable as it is a retrospective study

Heavy smokers reported considerably higher pain intensity compared to non‐smokers in the studies of Melis et al.^24^ and Weingarten et al.^26^ Melis et al.^24^ reported greater pain intensity in smokers as compared to non‐smokers. In addition, they also reported a positive correlation between the dose (numbers) of cigarettes and pain severity, which was more pronounced in the female patients. Weingarten et al.^26^ reported that current tobacco users experienced greater pain intensity. The authors also found functional impairment among tobacco users, with pain interference seen in daily activities affecting social life and work.

The relevance of psychosocial factors to TMD pain and impairment was investigated in the study of Sanders et al.^25^ The study discovered that psychosocial stresses and coping techniques had a substantial impact on TMD pain intensity and impairment levels, even if tobacco smoking was not the main emphasis. These results can be linked to tobacco usage because smoking is frequently used as a stress‐reduction technique. According to Weingarten et al.,^26^ psychological discomfort, such as anxiety and depression, is frequently more prevalent in TMD patients. Psychosocial stressors have a major effect on TMD, especially in those who have a myofascial component. The authors reported that smokers experience more psychological distress than non‐smokers, and this anxiety is linked to worse TMD clinical aspects.

### 3.4 Metanalytic results

**FIGURE 2:**
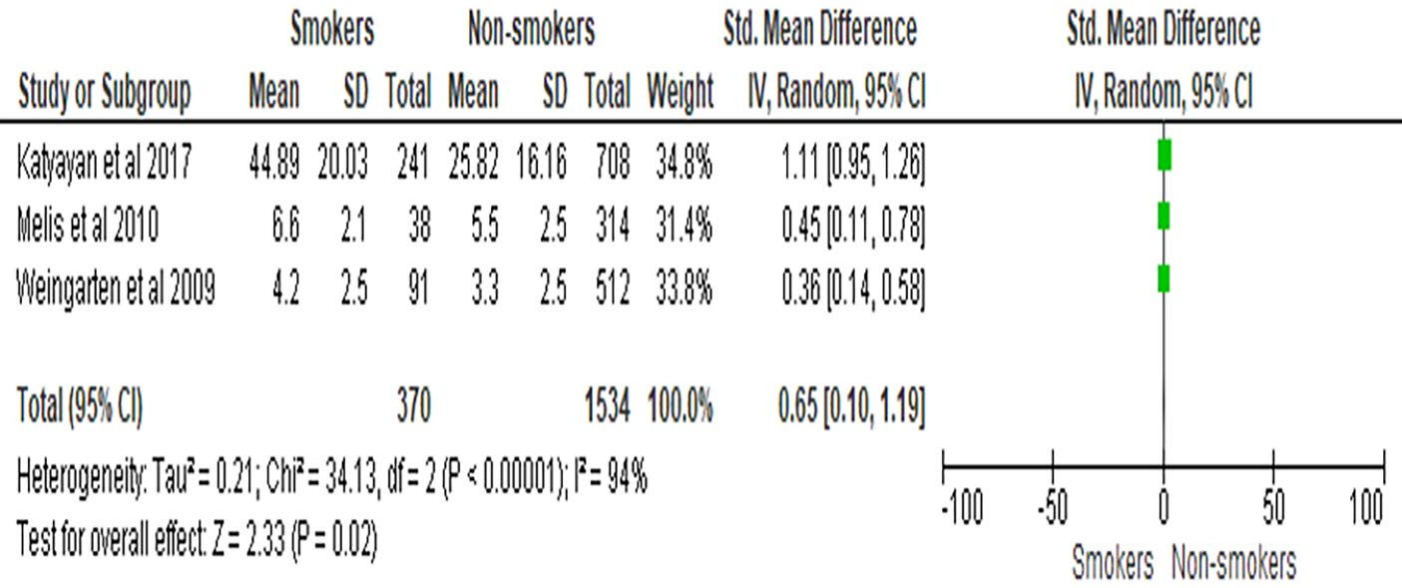
Comparative evaluation of TMD pain intensity between smokers group and non‐smokers group.

**FIGURE 3:**
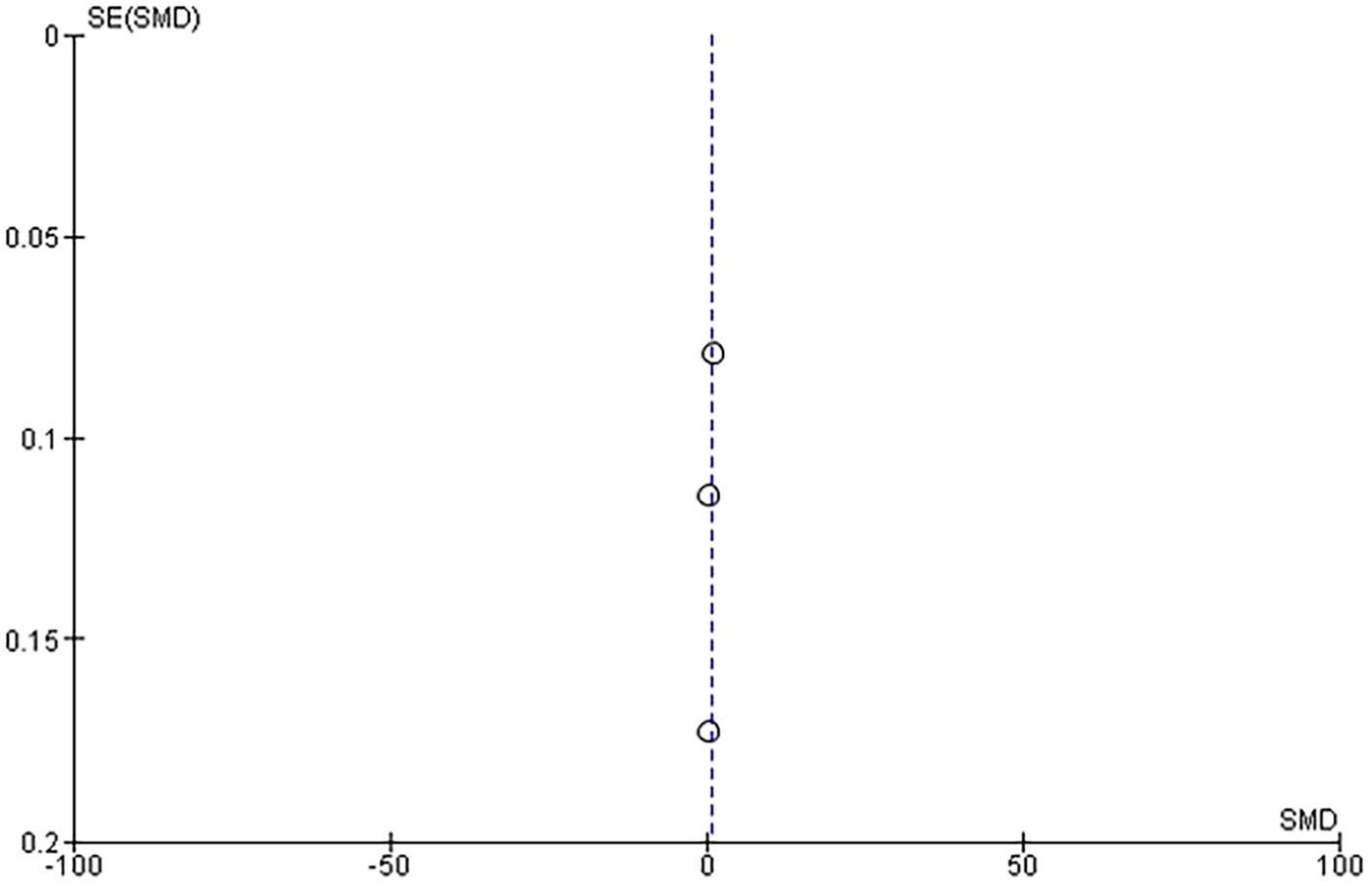
Publication bias for TMD pain intensity between smokers group and non‐smokers group.

The meta‐analysis included three studies comparing TMD pain intensity between smokers and non‐smokers. The forest plot illustrates the effect sizes and confidence intervals for each study, with squares representing the study‐specific effect sizes and horizontal lines indicating the 95% confidence intervals. The overall meta‐analysis result showed that TMD pain intensity was higher in the smokers group compared to the non‐smokers group, with a weighted mean difference (WMD) of 0.65 (BPM) (95% CI: [0.10, 1.19], *p* = .02). Heterogeneity among the studies was assessed using the I2 statistic, which was found to be I2 = 94%, indicating high heterogeneity as seen in Figure 2. Publication bias was assessed using a funnel plot, which indicated potential symmetry as seen in Figure 3.

### 3.5 Risk of bias assessment

All the studies assessed as per the NOS were scored 7. None of the studies were age‐ and gender‐matched between smokers and nonsmokers, thus reducing scores in the selection domain. As Research Diagnostic Criteria for Temporomandibular Disorders (RDC/TMD) was used in all studies, no bias was noted in outcome domain.

## 4 DISCUSSION

The findings of this systematic review showed that a definite association of impact of smoking on TMD pain and intensity. Tobacco smoking has the potential to aggravate TMDs through a combination of behavioural and biological causes. In the temporomandibular joint and related musculature, nicotine and other compounds in tobacco smoke can decrease blood flow, impair tissue oxygenation and promote inflammation. These effects can worsen discomfort and impede the healing process. In addition to its established effects on immunity, smoking may make one more susceptible to infections and inflammation, both of which can exacerbate the symptoms of TMD. In addition, smoking can cause bruxism or teeth grinding, which is a condition that puts extra strain on the temporomandibular joint. Another concern is psychological stress, since stress is known to exacerbate the discomfort of TMDs, and smoking is frequently used as a coping method. The confluence of immune response modification, behavioural factors and physiological stressors accounts for the increased pain sensitivity and functional impairment that are seen in TMD smokers.^22^, ^23^, ^24^, ^25^

Anxiety and depression are frequently seen in TMD sufferers. In addition to exacerbating pain perception, psychological anxiety can play a role in the development and maintenance of TMD symptoms. Anxiety and stress can result in habits like bruxism, which grinds teeth, aggravating tension‐related headaches. Smoking is a common coping strategy adopted by people, who are depressed or anxious. Smokers are more likely to experience psychological discomfort at higher levels, which may have an impact on the onset and severity of TMD. TMD symptoms might be greatly increased when mental health problems and smoking coexist. Smokers who additionally struggle with depression or anxiety are more likely to develop severe TMD discomfort and functional impairment.^24^, ^25^

Smokers with chronic pain frequently report increased pain intensity, increased functional impairment, increased anxiety and depression, and sleep difficulties.^27^, ^28^, ^29^ According to a study, smokers with TMD are even more likely to experience side effects from treatments.^10^ Contrarily, another evaluation of individuals receiving multimodal care for chronic pain discovered that smokers had equivalent or even superior immediate therapeutic effects compared to non‐smokers, thus complicating the impact of smoking on the severity of TMD pain and treatment outcomes.^11^, ^30^, ^31^

Smoking may have an impact on pain through a variety of processes, some of which may include allergic or inflammatory pathways. Smoking is known to block anti‐inflammatory cytokines while causing pro‐inflammatory cytokines to be produced.^32^, ^33^, ^34^ It increases the permeability of the respiratory epithelium and intensifies allergen sensitivity. Additionally, smokers have higher serum levels of immunoglobulin E, which decline with age. The molecular pathways through which smoking may affect the severity of chronic pain have been the subject of several studies.^35^, ^36^, ^37^, ^38^

Despite limited awareness, substantial epidemiological and clinical evidence underscores the plausible association between smoking and TMD‐associated pain, mirroring similar links established between tobacco use and diverse pain manifestations. Notably, the pivotal finding of this investigation highlights that the CS group across the selected papers^23^, ^24^ exhibits elevated TMD pain intensity and manifests less favourable responses to treatment in contrast to their NS or FS counterparts. This empirical evidence lends credence to the hypothesis that smoking may inherently potentiate pain perception and reporting, corroborating insights from previous research that delineates a positive correlation between smoking and heightened pain intensity across various patient populations.^39^, ^40^, ^41^, ^42^, ^43^

For instance, an investigation by Yunus et al.,^44^ which judiciously controlled for factors such as education and age, identified a notable positive relationship between smoking and pain among patients grappling with fibromyalgia. This finding aligns with a broader body of research linking smoking with augmented pain intensity in clinical contexts, further accentuating the potential role of smoking in modulating pain perception. However, nuances emerge in the assessment of pain intensity in smoking and nonsmoking TMD patients, as elucidated by the study conducted by Weingarten et al., which was included in our review.^26^ This research indicated that pain intensity, as gauged by the graded chronic pain scale—an amalgamation of pain intensity and its resultant interference in daily life—no longer exhibited statistically significant differences between TMD patients who smoked and those who did not.

Collectively, these findings underscore the intricate interplay between smoking and pain perception, substantiating the need for comprehensive exploration of this association within the specific context of TMD. Such investigations carry implications not only for elucidating the underpinnings of pain in TMD patients but also for advancing our understanding of the broader mechanistic links between smoking and diverse forms of pain, ultimately paving the way for more targeted interventions and therapeutic strategies.

However, it is essential to acknowledge the limitations inherent in the methodology and scope of this systematic review. First, one significant limitation is the potential for publication bias. Systematic reviews rely on published studies, and there is a possibility that studies with negative or null findings regarding the association between smoking and TMD may not have been published or may not have been included in this review. This bias could influence the overall conclusions of the study. Second, the variability in methodologies and populations across the included studies is a limitation. The studies encompassed diverse regions, sample sizes, and follow‐up periods, which can introduce heterogeneity into the synthesis of findings. This heterogeneity may limit the ability to draw uniform conclusions about the relationship between smoking and TMD.

## 5 CONCLUSION

The results of our review suggest that individuals who smoke tobacco are more likely to experience higher levels of pain intensity associated with TMDs when compared to non‐smokers. This finding underscores the detrimental impact of tobacco smoking on TMD‐related pain, highlighting the need for greater awareness and intervention in this population. Further research, including longitudinal studies and investigations into the underlying mechanisms, is needed to deepen our understanding of the relationship between tobacco smoking and TMD pain intensity.

## CONFLICT OF INTEREST STATEMENT

The authors declare no conflict of interest.

## Data Availability

The data will be available on reasonable request from the corresponding author.
